# Preventive pharmacological treatment in subjects at risk for fatal familial insomnia: science and public engagement

**DOI:** 10.1080/19336896.2022.2083435

**Published:** 2022-06-23

**Authors:** Gianluigi Forloni, Ignazio Roiter, Vladimiro Artuso, Manuel Marcon, Walter Colesso, Elviana Luban, Ugo Lucca, Mauro Tettamanti, Elisabetta Pupillo, Veronica Redaelli, Francesco Mariuzzo, Giulia Boscolo Buleghin, Alice Mariuzzo, Fabrizio Tagliavini, Roberto Chiesa, Anna Ambrosini

**Affiliations:** a Department of Neuroscience, Istituto di Ricerche Farmacologiche Mario Negri IRCCS, Milano, Italy; b Ulss 2 Marca Trevigiana Ca’ Foncello Hospital, Treviso, Italy; c AFIFF, Treviso, Italy; d Fondazione IRCCS Istituto Neurologico ”Carlo Besta”, Milano, Italy; e Fondazione Telethon ETS, Milano, Italy

**Keywords:** clinical trial, disease incidence, genetic prion diseases, patient engagement, tetracyclines

## Abstract

Engaging patients as partners in biomedical research has gradually gained consensus over the last two decades. They provide a different perspective on health priorities and help to improve design and outcomes of clinical studies. This paper describes the relationship established between scientists and members of a large family at genetic risk of very rare lethal disease, fatal familial insomnia (FFI). This interaction led to a clinical trial based on the repurposing of doxycycline – an antibiotic with a known safety profile and optimal blood–brain barrier passage – which in numerous preclinical and clinical studies had given evidence of its potential therapeutic effect in neurodegenerative disorders, including prion diseases like FFI. The design of this trial posed several challenges, which were addressed jointly by the scientists and the FFI family. Potential participants excluded the possibility of being informed of their own FFI genotype; thus, the trial design had to include both carriers of the FFI mutation (10 subjects), and non-carriers (15 subjects), who were given placebo. Periodic clinical controls were performed on both groups by blinded examiners. The lack of surrogate outcome measures of treatment efficacy has required to compare the incidence of the disease in the treated group with a historical dataset during 10 years of observation. The trial is expected to end in 2023. Regardless of the clinical outcome, it will provide worthwhile knowledge on the disease. It also offers an important example of public engagement and collaboration to improve the quality of clinical science.

## Introduction

Fatal familial insomnia (FFI) is an ultra-rare autosomal dominant neurodegenerative disease clinically characterized by sleep disruption, alteration of the sleep/wake rhythm, dysautonomia, and motor signs, with thalamic degeneration. Genetically, the disease is associated with aspartic acid (D) to asparagine (N) mutation at codon 178 coupled with methionine (M) at the polymorphic codon 129 of the *PRNP* gene encoding the prion protein (PrP). The penetrance of the disease is very high and information on its natural history is limited [1]. No therapeutic options are available, and patients can only be offered palliative care, including management of the neuropsychological symptoms [2].

Our group is conducting a long-term preventive clinical trial on individuals at genetic risk of FFI, repurposing the small molecule doxycycline [3], with the support of Fondazione Telethon (hereafter Telethon), the main Italian research funding agency in the field of genetic diseases [4]. The study started in 2012 and is planned to last 10 years. Its implementation and conduction presented challenges at different levels, some typical of ultra-rare diseases, such as the small number of patients and the scarcity of documented knowledge on the natural course of the disease. These aspects had a strong impact on the trial design and its statistical power. Other issues are specific to neurodegenerative diseases that occur in adults, with clinical manifestations when neuronal cells are already too compromised to expect any reversal of the pathological molecular processes, or at least physiological stabilization. As a consequence, only preventive studies have any chance of efficacy, but, due to the wide range age at disease onset and the lack of prodromal biomarkers [5], deciding when to start treatment is largely a gamble. Moreover, the complexity of the study, its ethical implications, and the long-term commitment of participants imply close alliance with the participants, which can only be based on good communication and close listening, as well as recognition of the different perspectives and priorities.

Here, we analyse the process of developing and managing the trial from the view points of the stakeholders: the members of a large family at genetic risk, organized in an association, the clinicians and other professionals, and the funding agency. We believe other patient/research groups may encounter similar challenges; therefore, sharing our experience and offers support for ongoing/future initiatives.

## Historical perspective

### Discovery of the disease

The investigators involved in this study learned of the existence of an Italian group of individuals at genetic risk for FFI by chance, reading a lay interview of Dr Ignazio Roiter, director of general medicine in Treviso Hospital, and specialist in endocrinology. As a young physician, he stumbled upon a mysterious neurological disorder that affected in sequence three members of his spouse’s family. In middle age, they developed strange neurological symptoms, insomnia and other neurovegetative signs, with rapid disease progression leading to death in a few months. He realized this was a new disorder of genetic origin with insomnia as a central symptom rather than a consequence of other neurological alterations. The sleep disturbances and several other neurological and neurovegetative symptoms were confirmed by a tertiary neurological centre in Bologna that Dr Roiter consulted, and post-mortem brain analysis showed marked neuronal depletion and astrogliosis restricted to the anterior and dorsomedial thalamic nuclei. Thus, the first case of fatal familial insomnia, as the new disease was named, was described in the literature [6]. In collaboration with the group in Bologna, Dr Roiter also described the hormonal alterations in FFI subjects [7]. Successively, the disease was recognized as part of the prion disease group, also known as transmissible spongiform encephalopathies (TSE) [8,9].

Emphasis was laid on the molecular genetic investigation, which had brought to lightthe PrP D178N mutation associated with a form of genetic Creutzfeldt–Jakob disease (CJD) or FFI depending on the polymophism at codon 129 where either valine (V) or M can be found [9,10]. The 1990s were a period of exceptional scientific growth for prion diseases, culminating with the Nobel Prize to Stanley Prusiner in 1997 for discovering a new mechanism of disease transmission, involving self-replicating proteins, which he named prions, composed of an abnormal isoform of PrP, commonly referred to as PrP^Sc^ [11,12].

### An Association seeking scientific interest

Dr Roiter became the referral physician for the large FFI family group, with the support of Dr Artuso, a primary care practitioner involved in the medical assistance, sharing information about the disease and encouraging the creation in 2002 of an association to support the families involved and promote research on FFI and other prion diseases (Fatal Familial Insomnia Family Association, AFIFF, http://www.afiff.net). A special feature of this association was that the core group was made up of healthy people who shared the risk of an extremely rare, fatal disease, with no other patients, no relatives, no healed patients – only ‘normal’ people with a sword of Damocles over their heads. Like with other ultra-rare diseases, living with this, in the absence of any remedy, is not easy and biomedical research becomes the only way to manage the daunting prospects.

The concept that a reasonable number of people were required to form a credible subject of investigation influenced the contacts with clinicians, who provided support and assistance but could offer no prospects for the future.

When in 2003 the scientists met Drs Roiter and Artuso and the FFI Association in Treviso, the immediate impression was a mixture of willingness to participate in the research and a genuine request for scientific help. In a sense, the Association members recruited ‘their’ scientists, identifying common ground and interests.

The progress in prion biology knowledge and the contribution of basic research to the understanding of prion disease pathogenesis were illustrated to the audience in Treviso, admitting that no treatment was available. In the discussion that followed, the Association put challenges to the scientists, questioning the real health impact of their studies.

Biological material, historical data and any other useful information from their experience were made available for scientific projects aimed at clarifying the disease pathogenesis and developing possible therapeutic approaches. Genotyping the large family to identify carriers of the mutation followed, although the clinicians became immediately aware that none of the members had known their own genotype. Participants were willing idea to contribute blood or tissue specimens for a better understanding of the disease and – hopefully – to find a cure, evading the awareness of the inevitable fate. Consequently, the group of genotyped people remained homogeneous with no distinction between carriers – one quarter, and non-carriers – three quarters of the members. This sort of ‘herd defense’ had practical and ethical implications; the main point was the impossibility of arresting transmission of the disease by interventions in the reproductive phase, such as in vitro fertilization with preimplantation genetic testing. It is true that identification of the carriers could break the solidarity bond within these families separating the fortunate from the unfortunate. The FFI mutation is highly penetrant, as shown by Dr Roiter’s analysis [13] of the 46 cases in the Italian kindred, which indicated that only two carriers could be considered ‘escapers’, with a penetrance of 95%. Essentially, the mutation is almost always accompanied by the disease. This partially explained the desire of the AFIFF members to be blind to their own genotype. Cultural differences also play an important role in the decision-making as we had the opportunity to verify with members of the American CJD Foundation (https://cjdfoundation.org). The CJD Foundation organizes sections where scientists can talk directly to members of the Association and it is not unusual to meet people who spontaneously declare their status as carriers of *PRNP* mutations and ask for news in the field. Recently, a similar attitude has started to penetrate the Treviso community too, with the younger members of the FFI family considering the possibility of knowing their own genotype and the biotechnological support available to avoid transmission of the disease to their offspring. Since the decision to know their own FFI genotype can have consequences on the entire community of participants, they agreed to postpone that knowledge to the end of the trial. Promoting genetic counselling and scientific information also requires an effective psychological support to provide members of this family at genetic risk with sufficient help to make informed decisions and plan for their future. Periodic meetings were organized with trial participants and the other members of the AFFIF to provide updates on the study and illustrate scientific news in the field.

## Clinical research directions

### Genetic and clinical characterization

To establish the genotype in relation to the FFI mutation, nearly 100 samples were collected among the members of the Treviso families. Twenty were found to be carriers of the D178N mutation but, as specfied in the consensus they had given, their genotype was not revealed to them. In a limited number of subjects, carriers and non-carriers of the mutation, a small skin biopsy was taken from the shoulder to establish fibroblast cell cultures. This served to develop a human-derived cell model to investigate the influence of the mutated form of PrP on cell functions and these cells were used to confirm the results in transfected cell lines [14].

### Towards a pharmacological trial

The idea of design an intervention study on FFI took form after in vitro and in vivo experiments confirmed that doxycycline could disrupt or inhibit the formation of prion protein aggregates associated with pathological events [15–17]. Doxycycline is a well-known antibiotic of the class of tetracyclines, with a good safety profile after chronic treatment, and ability to cross the blood–brain barrier [18]. Its heterocyclic structure with a planar conformation interferes with beta-sheet amyloid formation of the prion protein [19,20]. Additional beneficial effects in vivo may be due to its antioxidant and anti-inflammatory properties, as recently shown [21]. The drug’s neuroprotective effects have been reported in different animal models of neurodegenerative disorders [22–24].

Between 1996 and 2004 doxycycline was offered as open compassionate treatment to patients with CJD diagnosed at the ‘Carlo Besta’ Institute in Milan and retrospective analysis showed an increase of survival time [25]. Similar results were later obtained in an independent observational study in Germany [26]. However, a randomized clinical trial against placebo to test the efficacy of doxycycline in CJD patients, coordinated by Dr Tagliavini started in 2007 [27] but gave negative results, probably due to the advanced stage of disease in recruited subjects.

As discussed with the Association members, all these findings, including the substantial absence of side effects, concurred to select doxycycline as a good candidate for FFI carriers and stimulated interest in a preventive clinical trial. The previous studies indicated that chronic treatment with 100 mg/day was well tolerated and suitable to engage targets beyond the antimicrobial activity [27–30]. As reported by Lucchetti et al. [18], the cerebral levels of doxycycline after a treatment with 10–100 mg/kg daily in mice, exerting neuroprotective activities, are comparable with the cerebral concentration of doxycycline observed in humans after chronic treatment with 100 mg daily [27].

Working on the study design was extremely complicated. On the one hand, there were difficulties due to the lack of biological markers to monitor the efficacy of treatment and disease progression, combined with the obvious scarcity of participants. On the other hand, the disease is so aggressive that arresting its progression at the onset was extremely unlikely, and prolonging survival in disease conditions was not desirable either. As in many other conditions, treatment in the presymptomatic period increased the possibility of interfering with the pathogenesis.

A preventive approach was preferable which, if effective, could prolong health or even hinder the development of the disorder. The best possible protocol was based on historical data and statistical analysis, and practical questions were discussed with the members of the AFIFF and potential participants.

The main issues concerned the selection of participants and at what age to start treatment. Historical data indicated a distribution of FFI mortality peaking around the age of 53, and death generally before 60 years, with only two surviving carriers after this age. On the basis of genetic data of the individuals belonging to this large family group and their ages, it was decided to select a group of ten carriers in the age range 44–53 years, to be treated for 10 years. The age limit of 44 years to be enrolled was considered to guarantee the minimal exposure to treatment in order to appreciate its effect.

In addition to their important contributions to the study design, discussion of the protocol with the Association members indicated again their desire not to be informed of their own genotype. Respecting this position had substantial consequences in how the study was organized and led to recruitment of carriers and non-carriers on the basis of their age, with carriers receiving doxycycline and non-carrier placebo, as identical tablets. A grant application was submitted to Telethon in 2010 and funding was approved.

### The trial protocol

**Figure 1.**
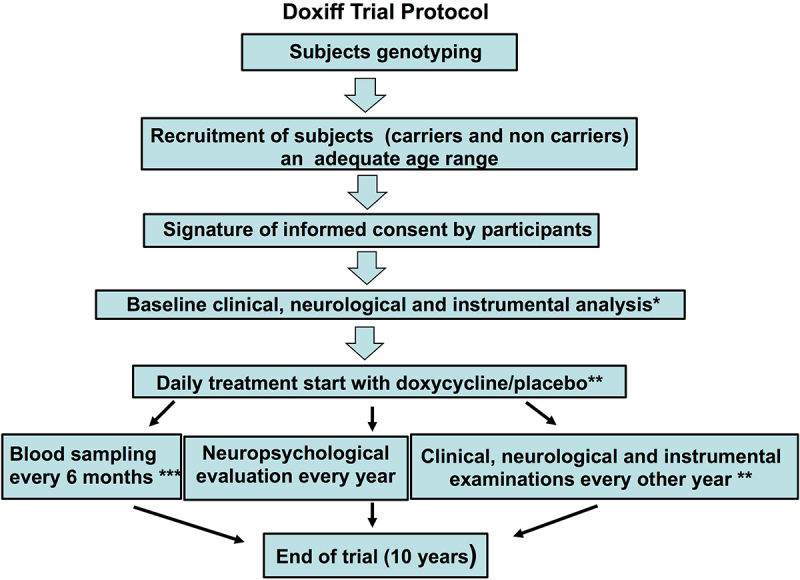
Flowchart of the clinical trial protocol.

The trial protocol (DOXIFF) of the study was reported in The National Monitoring Centre on Clinical Trials (OsSC) operating under the control of the Italian Medicines Agency (Eudra CT 2010–02223328) and published [3, Figure 1]. Case history and symptomatology took into account the patient’s lifestyle, previous and current illnesses, the use of prescribed medicines and stress-inducing factors. The general objective examination showed the health status and indicated how prone the patient was to common illnesses. Inclusion criteria: subjects aged 44–53 years; no conditions known to be contraindications to the use of tetracyclines; written informed consent. Exclusion criteria: end-stage liver, heart or renal disease, active malignancy, pregnant or lactating women [3]. Ten subjects carrying the mutation were treated daily with 100 mg doxycycline hyclate (Bassado), and 15 non-carriers received a placebo.

*Selected subjects underwent of a full neurological examination including at baseline and at follow-up visits: (1) mental status and cognition (level of alertness, attention and cooperation, orientation, memory, language, calculations, right-left confusion, finger agnosia, agraphia, apraxia, neglect, sequencing tasks and frontal release signs, logic and abstraction, delusions and hallucinations, mood), (2) cranial nerves, (3) motor and sensory functions, (4) reflexes, (5) coordination and gait. The polysomnographic recording was done according to internationally accepted technical standards [54,55]. The specific variables monitored and recorded during polysomnographic assessment were global neural electroencephalographic activity, eye movements, submental electromyographic activity, heart rhythm, respiratory effort, nasal/oral airflow, oxygen saturation, body position and limb movements.** According to the approved amendment from May 2019 the doxycycline dosage was doubled, to two 100 mg tablets daily instead of one.*** The laboratory tests include fasting blood sugar, glycated haemoglobin, transaminase (SGOT and SGPT), direct and indirect bilirubin, gamma glutamyl transferase, alkaline phosphatase, albumin, prothrombin time, creatinine, BUN, full urine test, full cholesterol levels, HDL-cholesterol, triglyceride, complete blood count, protein electrophoresis, erythrocyte sedimentation rate and C-reactive protein. The plasma levels of cortisol, FT3 and FT4, free testosterone, 17 beta oestradiol, progesterone, 17 OH progesterone, DHEA-s, plasma renin activity and aldosterone and IGF-1 were investigated. The integrity of the hypothalamus-pituitary axis and correct response of the complex neuroendocrine network was determined by the response of the GH after GHRH+arginine test.

**Figure 2.**
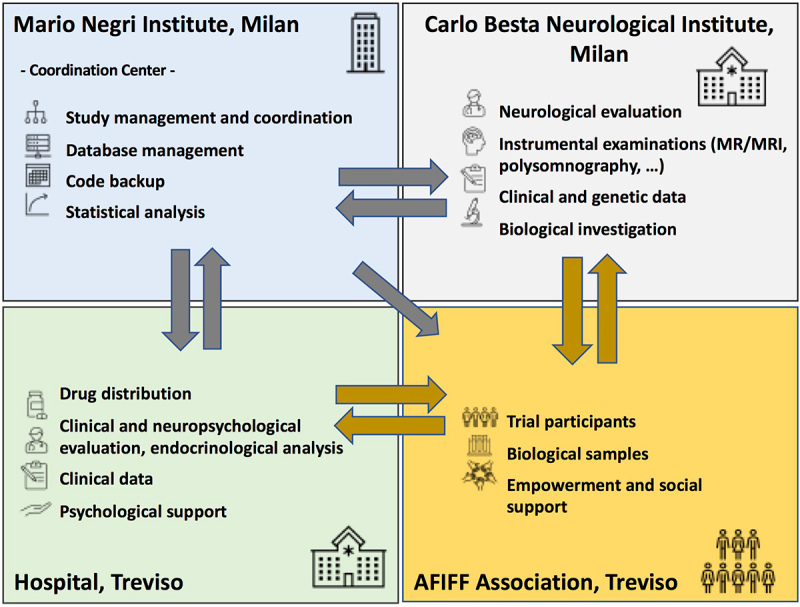
The interactions among partners in the preventive clinical trial with doxycycline in subjects at risk of fatal familial insomnia. Each box lists the activities of each partner, the centre in charge and its interactions with the other partners, and the communication flow.

FFI is a very rare disease and although we had a favourable condition thanks to the Association members, the number of subjects recruited and the long duration of observation did not recommend further division in the group of carriers, while the non-carrier group was included only to satisfy the request of participants to be blind about their own genotype. All participants underwent clinical and instrumental evaluation at recruitment before starting treatment, and every second year after that, with blood sampling for laboratory tests and endocrinology every 6 months, and neuropsychological examinations every year. These analyses were established on the basis of previous investigations and were done by personnel blind to the FFI genotype of the subjects (Figures. 1 and 2).

Statistical analysis considered the survival curve based on historical clinical data, with the probability of survival for each subject calculated on their age at the date of inclusion in the study. Different scenarios were drawn, depending on the final number of carriers enrolled. When the trial was proposed to potential participants, a few, including three presymptomatic carriers, said they preferred not to participate, in view of the scant evidence of doxycycline’s efficacy in FFI and the long-term commitment required. This refusal would have had a critical impact on the overall feasibility of the study, but new subjects were identified among members of the family living outside the Treviso area, who at that time had less contact with the original group. In all, at the closure of the enrolment phase, ten carriers were included in the study. With these numbers, however, the cumulative probability of observing up to three cases in 10 years is lower than 0.05; therefore, if fewer than four cases develop the disease, the trial will be considered successful in demonstrating the efficacy of doxycycline.

In order to draw this conclusion, we then calculated the probability of zero new cases in 10 years in the group of ten subjects, and then the probability of one, two or three new cases. When these probabilities were added together, the cumulative probability was lower than 0.05, while the inclusion of four cases raised the cumulative probability to a value higher than 0.10. Therefore, a cut-off of three new cases was established as a positive outcome of the trial. A control group of carriers was not taken into consideration due to ethical issues, but the refusal of a few subjects to participate will offer the possibility at the end of the trial to make an external anecdotal comparison (this is not part of the trial protocol).

The start of the trial (budget and ethics board approvals, drug and placebo availability, trial database, etc.) and the recruitment phase took a long time. The first treatment started in the second part of 2012 and the enrolment phase closed in April 2013 with 25 participants. The Data Safety Monitoring Board surveyed the course of the study and addressed any ethical implication at its periodical meetings.

A few amendments had to be made to the protocol. For instance, to take into account the potential inconvenience that any minor side effects of doxycycline could reveal the carrier status, given the fact that participants are part of the same large family group and share information, it was established that the non-carriers too periodically received doxycycline for a limited time in a random schedule. Other amendments included the decision taken with the support of the Data Safety Monitoring Board after the first case of manifestation of the disease and the inclusion of biological exploratory studies based on new research evidence (see below). The manifestation of a second case and re-examination of the safety profile of doxycycline in an interim analysis suggested the daily drug dosage could be doubled; an amendment was approved, and as of May 2019 carriers in the study received two 100 mg tablets of doxycycline daily. The study is expected to be completed by 2023.

## Biological Investigations

Besides the therapeutic opportunity, the preventive study gave the investigators the unique possibility to follow FFI mutation carriers longitudinally in an asymptomatic phase and characterize the biological changes associated with the mutated form of prion protein. The fibroblasts from skin biopsies of carrier and non-carrier members of the FFI enabled us to develop an experimental model to investigate the cellular alterations associated with D178N/129 M prion protein mutation. During the progression of the trial Dr Artuso proposed to the scientific committee the possibility of testing FFI at-risk subjects with a new diagnostic tool in protein misfolding. The approach, originally proposed by Gianluigi Zanusso and Byron Caughey [31,32], was based on determining the presence of PrP^Sc^, an unquestioned marker of the disease, in olfactory mucosa taking epithelial cells whit a nasal swab, a minimally invasive procedure. The PrP^Sc^ identified by an amplification system (real-time quaking-induced conversion, RT-QuIC or Protein Misfolding Cyclic Amplification, PMCA) should indicate activation of the pathological process in a presymptomatic phase[33,34].

The application of these high-sensitivity techniques on epithelial cells from the nasal mucosa is particularly important because of the direct connection between this tissue and the brain. PrP^Sc^ must be identified in asymptomatic conditions to activate a therapeutic strategy in an early phase of the disease and, in sporadic events, to clarify a suspected diagnosis of TSE. During a plenary session with the participants, the brushing biopsy technique was illustrated with a video and the significance of the investigation was discussed to address any issues or doubts. An amendment to the study protocol was prepared, with the necessary consensus form, and this part of the study was run by the investigators at the Besta Institute in Milan. The initial results of the first brushing biopsy campaign confirmed the positive findings only in affected FFI cases [35].

The biological material was also used in an international collaboration for the analysis of anti-PrP antibodies in plasma of subjects with prion diseases or carriers of PrP mutations associated with familial forms of TSE. This study, coordinated by Dr Adriano Aguzzi from Zurich, was inspired by the immunotherapeutic approach to Alzheimer’s disease: the principle was that the progression of neurodegenerative disease might be controlled by the body’s immunological reaction. The last- generation anti-β-amyloid antibody, aducanumab, was purified from fluid from an almost centenarian individual with no cognitive decline [36]. Apparently, these antibodies raised a protective barrier against the β-amyloid mediated toxicity. Aguzzi’s study therefore aimed to determine the presence of autoantibodies against the prion protein in carriers of a genetic prion disease mutation and controls, and whether this influenced the development of the disease.

This investigation, which also used small blood samples from carriers and non-carriers was discussed with participants, during a general meeting, a teleconference was organized with Dr Frontzek from Zurich, who illustrated the study. The therapeutic perspective was carefully assessed in terms of possible biotechnological development of an anti-prion treatment based on the purified antibody associated with the specific pathological condition.

As recently reported [37], a large number of samples was analysed, but the presence of autoantibodies against the prion protein was not influenced by the prion mutations. On the other hand, the high anti-PrP titre, found occasionally, did not suggest any correlation with the disease. The lack of association between anti-PrP autoantibodies and the physiological or pathological condition did not rule out the possibility that immunotherapy with the anti-PrP antibody might achieve some control of the disease, as proposed in several experimental conditions [38,39]. However, this research did not take specific directions along this line.

Another collaborative project was proposed by Dr Chiesa together with Dr Gustavo Mostoslavsky and Dr David Harris of Boston University, to generate induced pluripotent stem cells (iPSCs) from peripheral blood mononuclear cells from carriers of the FFI mutation (with CRISPR-corrected isogenic lines) and non-carrier relatives, using a well-established reprogramming protocol [40–42]. This collaborative project has three main aims: (1) To Differentiate iPSCs carrying the E200K or D178N/M129 mutations into neurons, astrocytes and microglia, and characterize the mutant PrPs in terms of their biochemical and cellular profiles; (2) To do genomic and transcriptomic analyses of iPSC-derived neurons, astrocytes and microglia carrying the E200K and D178N/M129 mutations; and (3) To determine the effects of the E200K and D178N/M129 mutations on synaptic structure and function, and test the ability of selected therapeutic compounds to rescue synaptic defects. The project was described to the participants who agreed to contribute, even if it had no directly impact on their condition. Among the participants, the level of interest in scientific issues tended to vary as expected, and was independent of individual cultural levels. However, there was firm confidence in the medical staff with appreciation for their efforts to provide adequate explanations for each and every step along the way.

## Psychological support plan

The need for specific psychological support emerged during a periodic meeting with the participants, after the start of treatment. As we mentioned, the participation in the trial on the one hand improved the health care monitoring of the subjects, but, on the other hand, it constantly reminded them of their potential condition as persons at risk for FFI. The need to work through this psychological state with the help of professionals led the trial coordinator to set up a psychological support plan, as requested by the participants. Telethon dedicated new funds and this programme was activated in 2013. The main purpose was to provide appropriate advice and to prepare the ground for dealing with the psychological impact of a negative event such as the onset of FFI in any of the individuals in the study.

Psychological support was designed 1) to give trial participants adequate personal support to address the burden of the disease risk; 2) to reinforce the participants’ compliance with the daily drug or placebo treatment and clinical analysis schedule in the protocol; and 3) to maintain and reinforce the participants’ motivation throughout the long observation period, to avoid anyone dropping for any reason.

Experience in this field was limited, and scant information was available about the psychological characteristics of subjects with genetic conditions at risk for a lethal disease and the willingness to participate in a clinical trial in a non-affected condition. The participants’ decision not to be aware of their own genetic condition was a further uncertainty factor influencing their psychological status over 10 years of observation and treatment. The long duration of the study with periodical examinations was itself another element requiring robust motivational support.

The experience of the European Huntington’s disease (HD) network was inspiring and highlighted the importance of synergy among people with a rare disease and their relatives, clinicians and researchers and the need for them to work together to advance research and improve the quality of care(http://www.ehdn.org) [43] The HD working group also addressed the psychological needs related to diagnosis and/or predictive testing for HD, two clinical services with different purposes that raise very different ethical issues and develop important recommendations for genetic counselling within families [44,45]. Interestingly, previous studies on a self-selected group discussing preventive testing for HD showed significantly greater mean ego strength and significantly better coping strategies than the general population; test participants reacted with active problem solving, with comforting and optimistic thought, and with social support seeking. Among the test applicants, 15% had at least a mild depression on the Beck Depression Inventory and/or a high score for general anxiety on Spielberger’s anxiety scale during the pretest period [46].

The European HD Network also indicated that the subject’s social network may act as a protective factor. Indeed, a supportive and understanding relationship in childhood and close personal relations later in life reduced the effect of childhood stressors on adult psychological functioning [47].

In line with these indications, the psychological support programme of the trial was organized with a systemic relational approach in a familial context [48], with particular attention to individual relational skills [49]. As the clinical study progressing with, it became even clearer how important was the role the scientists in influencing the of participant attitudes, especially their confidence in the project. Despite their distinct roles and functions, the close relationships with the participants involved scientists and physicians in the whole system, positively influencing the study.

The psychological intervention was organized as individual or couple interviews, in family or group sessions (Table 1). The psychological programme involved about 80 people per year, and according to their individual, couple, family and group interpersonal skills, one or two contacts have been made each year.

**Table 1.** Psychological support programme: levels of interventions by sessions.

Level of intervention	First period	Second period	On request	On appearance of FFI
Single participant (individual)	X		X	X
Single participant and partner (couple)	X		X	X
Single participant with her/his nuclear family (family)	X		X	X
Sibling participants and their nuclear families (extended to family of origin)	X			
The whole group of participants and their families, and researchers	X	X		X
The group of participants		X		
The group of offspring of the participants		X		

The first period (2013–2016) was dedicated to consolidating the relationships between family members. Meetings of several families of participants have intensified the knowledge and relational bonds in the whole relational FFI group (Table 2).

**Table 2.** Psychological support programme: aims.

Aim	First period	Second period	On request	On appearence of FFI
Consolidating the relationship between family members of participants and within the families of origin	X			
Promoting the knowledge and relational bonds in the entire relational FFI group	X			
Motivating and Involving to participate in the study	X	X		
Promoting awareness and fostering the initiative of belonging to the research group		X		
Preparing for the exit and acceptance of the study before moving to genetic counselling		X		
Psychological support	X	X	X	X
Accompaniment at the end of life				X
Working through mourning		X		X
Continuous monitoring of the participants and their families, with self-report instruments	X	X		

Subsequently, the programme was extended to subgroups, particularly involving the second generation of offspring, a group of individuals who needed attention too, both in terms of psychological support and scientific information. Awareness of their own condition of potential carriers of the FFI disease mutation, but also as candidates for future investigations have grown over time, and an initial superficial acquaintance has become a solid relationship. The schedule of meetings in and between families has built up a network of links fundamental to maintain treatment compliance and to deal with the first negative episode among the participants.

Specific aspects of FFI in relation to a study in progress, the advantages of being part of an experimental project, the difficulties of managing a rare disease, and the prospects for the new generations were analysed. During the first period with no critical events, participation in the project was diligent but apparently passive, with limited emotional involvement and, underling the low level of problems, the psychologists defined this period as a ‘honeymoon’. The climate gradually shifted when the psychologists proposed topics like *living will*, but the fundamental turn came when the first FFI case in the trial was communicated to participants. In contrast with the expected negative consequence, the subjects boosted their solidarity, and developed proactive behaviour, they were – or wanted to be – more compact when facing any future adversities. For example, when the scientific committee of the study officially met the relatives of the first case a month after the patient’s death, several other participants asked to be present at that uncomfortable meeting to share the common sentiment of closeness.

After the appearance of FFI symptoms in a subject, assistance was extended to that person’s relatives, with continued support throughout the course of the disease, and help in the mourning process when the subject died. Similar support was provided to families of participants who presented symptoms potentially linked to FFI but subsequently recognized as due to other reasons.

The approach of the end of the trial raised several issues among the FFI Association members. First of all, their attitude towards genetic counselling changed, with expressions of interest of participants and their offspring for genotyping analysis. This change might be due to the prospect of new therapies and the possibility of access consulting programme to face medically assisted procreation. Undoubtedly, the levels of maturity, knowledge, and awareness of the participants and their families have grown considerably and the dialogue with the scientific staff on these issues has been and will continue to be extremely productive. This research effort illustrates the significant impact of partnership between patients and physicians-researchers on medical and scientific breakthroughs in a specific pathological field [50].

The initial goal of assisting in individual psychological well-being as the programme proceeds has evolved. Constant work to strengthen and repair relational ties between the participants had produced a group that is capable of interacting with the investigators and proposing solutions and common perspectives.

## The funding agency’s viewpoint

Telethon’s mission is to fund research on genetic diseases and their treatment, with particular interest in rare and ultra-rare conditions that are not attractive to industry [4]. Therefore, the agency and its review committee considered the proposal to address the unmet medical needs of this target population and run a pharmacological trial of great significance.

Despite the importance of the goals, the decision to finance it was not easy, for several reasons. First, the ‘classical’ trial design and evaluation methods used for non-orphan diseases could not be adopted here. A double-blind randomized approach was not applicable because of the ethical need to give individuals carrying the disease mutation a chance, while the lack of consistent natural history data to refer to and the low statistical power cast doubts on the feasibility of the study. This is typical of clinical studies in ultra-rare diseases, where the limited patient population is an obstacle to the collection of significant longitudinal data that contributes to knowledge of the disease, while also hindering the recruitment of adequate number of participants. Additional concerns were more specific to this study, namely the particularly long exposure to the drug, to establish its efficacy and clarify uncertainties about the ideal age at which to start preventive treatment.

These concerns were mitigated by evidence of the strong partnership of this established group of researchers with families and their doctors, which was considered reassuring at least from the perspective of adherence and long-term compliance of participants. Overall, this was seen as a unique opportunity to evaluate the therapeutic efficacy of a known drug for this devastating disease and the Board of Directors approved the commitment to support the trial. Normally Telethon finances extramural grants of up to 3 years, selected through competitive calls. This trial required a longer commitment and it had to be included as a special project in the Telethon portfolio. Even though it was not financed through a competitive call, periodic evaluations were scheduled every 3 years, to monitor progress and approve funds for the next 3 years. This allowed budget adjustments according to the actual numbers of subjects, the costs of clinical procedures, and any unforeseen costs, such as the psychological support. It also provided the investigators with helpful suggestions from peer reviewers to improve the clinical management of the study.

Over the years, the Telethon manager of the project has had opportunities to interact with the members of the FFI Association, the investigators and the other professionals involved, participating in building a strong sense of community among all stakeholders.

## Conclusions

The engagement of patients as partners in healthcare research has gained consensus over the last two decades [51,52]. A number of reasons support the inclusion of patients as active partners in biomedical research and encourage funding to promote this participation. First, it is ethically important to share medical and scientific information with the subjects of research, learning their point of view and promoting informed shared decision-making [53]. Moreover, patients can offer different perspectives in health priorities and contribute to improving the design and outcome of clinical studies. This paper describes the mutually beneficial interactions between scientists and the FFI Association, whose members from a large family at genetic risk of this very rare, and lethal neurological disease. This has been the key for the successful development of this preventive clinical trial with long exposure to pharmacological treatment.

A critical factor in the treatment of neurodegenerative disorders – and very likely one of the reasons for the numerous failures – is that a (potential) therapy is started at the clinical onset of the disease or later, when the neurodegenerative process is advanced and apparently no longer reversible. This concept has alimented biomedical research in two directions: the identification of early prognostic biomarkers to detect early pathological signs in subjects at genetic risk, and the design of preventive studies involving this population. The two directions are destined to converge in a single approach when treatment is provided in the preclinical phase and changes in biomarkers can be considered a reliable outcome.

In the absence of adequate tools, preventive trials can be designed with individuals at genetic risk of a specific disease and changes in its incidence is the only outcome measure. This is particularly complicated when facing a rare disease since only limited numbers of people are involved, information on the natural history of the disease is scarce and opportunities to find financial support are few. In these conditions, indispensable support can only come from people with a direct interest in investigating therapeutic approaches: the carriers of the genetic risk. The active engagement of the FFI Association was a key factor here to create the conditions for the preventive study, and their enthusiastic participation greatly helped overcome logistic and scientific obstacles. The availability of a drug with minimal side effects, for which there was a strong rationale, and the interest of an Italian funding agency, Telethon, whose mission is to find a cure for rare genetic diseases, were the other essential components that made the study scientifically sound and feasible. This partnership between participants and their association, the non-profit funding agency and the investigators generated an important synergy, which has already given positive effects in terms of human and scientific experience, even if the clinical outcome is not known yet.
